# Sleep-Disordered Breathing in Hospitalized Geriatric Patients with Mild Dementia and Its Association with Cognition, Emotion and Mobility

**DOI:** 10.3390/ijerph16050863

**Published:** 2019-03-09

**Authors:** Janine Gronewold, Robert Haensel, Christoph Kleinschnitz, Helmut Frohnhofen, Dirk M. Hermann

**Affiliations:** 1 Department of Neurology, University Hospital Essen, University of Duisburg-Essen, Hufelandstraße 55, 45147 Essen, Germany; janine.gronewold@uk-essen.de (J.G.); robert-haensel@gmx.net (R.H.); christoph.kleinschnitz@uk-essen.de (C.K.); 2 Department of Nephrology, Geriatric and Internal Medicine, Alfried Krupp Hospital Ruettenscheid-Essen, Alfried-Krupp-Straße 21, 45131 Essen, Germany; helmut.frohnhofen@krupp-krankenhaus.de; 3 Faculty of Health, Department of Medicine, University Witten-Herdecke, Alfred-Herrhausen-Straße 50, 58448 Witten, Germany

**Keywords:** sleep-disordered breathing, obstructive sleep apnea, dementia, cognition, emotion, mobility, sleep quality, geriatrics

## Abstract

Sleep-disordered breathing (SDB) is an emerging dementia risk factor. Data on the prevalence of SDB in dementia patients and its association with cognitive impairment is so far only based on patients with severe dementia. In 101 geriatric patients mostly with mild dementia recruited on German geriatric wards, SDB was assessed during overnight polygraphy in the patient room with a portable sleep apnea examination device and associations of SDB severity with severity of impairment in cognitive and emotional function as well as mobility were investigated. We also elucidated which factors influence compliance of SDB diagnostics. In 82 of the 101 dementia patients (81.2%), SDB could be assessed. Of those, only 12.2% had an apnea-hypopnea index (AHI) < 5/h demonstrating the absence of SDB. 40.2% exhibited 5/h ≤ AHI < 15/h representing mild SDB, and 47.6% revealed an AHI ≥ 15/h representing moderate/severe SDB. Patients in these three AHI categories did not significant differ from each other in demographical and clinical characteristics. Patients with an AHI ≥ 15/h particularly often presented with heart failure and vitamin D deficiency. We observed a low to moderate association between severity of SDB and severity of dementia. Tolerance of the nasal airflow sensor of at least 6 h was present in less than one third of all patients. The tolerant group exhibited more symptoms of depression and higher physical fitness compared to the non-tolerant group. We observed a high prevalence of SDB also in geriatric patients with mild dementia underlining the importance of SDB screening in the elderly.

## 1. Introduction

Sleep-disordered breathing (SDB) is an emerging dementia risk factor [1] but often remains undetected because patients report no subjective complaints like daytime sleepiness [2]. Although a causal relationship between SDB and dementia is not yet established, SDB is known to induce neurodegenerative changes as a consequence of sleep fragmentation and intermittent hypoxia [3]. In prospective population-based studies SDB was associated with increased risk for cognitive decline and dementia [4,5]. In small preliminary studies, positive airway pressure therapy, the gold standard for the treatment of obstructive sleep apnea, improved cognitive performance in dementia patients with SDB [6]. Such empirical evidence supports the hypothesis that SDB might be a reversible cause of cognitive decline and that treatment of SDB, especially in the early stages of dementia when patients are still largely independent, may slow dementia progression [7]. From a clinical perspective, it is thus important to know the prevalence of SDB in patients with dementia in order to plan resources and treatment. Even though a high prevalence of dementia [8] and SDB [9] has been consistently shown in the general elderly population, their overlap remains poorly understood. Only a few studies have analyzed SDB prevalence in dementia patients as well as the association between cognitive impairment and SDB in dementia. So far, research was mostly conducted by a single research group within the San Diego nursing home studies [10]. These studies showed that 70% of 235 institutionalized dementia patients showed SDB (defined by five or more respiratory disturbances per hour of sleep in portable sleep recording) and that SDB severity was significantly associated with dementia severity. Additional studies recruited smaller sample sizes and also included only patients with severe dementia of mostly Alzheimer’s pathology. In the majority of those studies, SDB severity was associated with dementia severity [10,11,12,13]. Only in one study including multiinfarct dementia patients, who were younger and exhibited lower levels of dementia than Alzheimer’s disease patients [14], and in a subgroup of patients with both cognitive impairment and depressive symptoms [12], SDB severity was not significantly associated with dementia severity. SDB detection and treatment would however be especially important in the early dementia stages since SDB is suggested to further decrease cognitive function via intermittent hypoxemia. Consequently, we analyzed the prevalence and severity of SDB in mild dementia patients treated in a German geriatric unit and investigated associations with severity of cognitive impairment and additionally with impairments in emotional function and mobility, which are highly prevalent in the elderly. Since SDB is often underdiagnosed and instrumental laboratory diagnostics especially of obstructive sleep apnea has been shown to be difficult in dementia patients, we also elucidated which factors influence compliance of nasal airflow sensor as a central part for the detection of obstructive sleep apnea.

## 2. Materials and Methods

### 2.1. Subjects

One hundred and one (101) patients (70% women, age range 66–97 years, mean 84.1 years and SD 6.5 years) with a dementia diagnosis according to ICD10 (F00-03) and Mini-Mental state examination test scores ≤27, were recruited on the geriatric wards of the Knappschafts-Krankenhaus in Essen, Germany between November 2015 and April 2016. The study was approved by the ethical committee of the medical faculty of the University Duisburg-Essen and all participants or their legal representatives gave written informed consent. 

### 2.2. Data Collection

#### 2.2.1. Sleep Disordered Breathing

SDB was evaluated during overnight polygraphy in the patient room with a portable sleep apnea examination device (ApneaLink AirTM, ResMed Germany Inc., Martinsried, Germany). Apnea-hypopnea index (AHI) was calculated as mean number of all apnea classes (unclassified, central, mixed, obstructive) and hypopneas per hour in the evaluation period. Apneas were defined as reduction of airflow to 0–20% that lasts 10 s or longer up to 80 s using an average of the last five breath cycles in accordance with the American Academy of Sleep Medicine (AASM) Manual for the Scoring of Sleep and associated Events [15]. Consistent with the AASM definition for the scoring of patients qualifying for positive airway pressure therapy reimbursement, hypopneas were scored when a flow reduction of at least 30% that lasts 10 s or longer up to 100 s was detected with a corresponding desaturation event of at least a 4% drop [15]. When no oximetry data was available, or was missing for a significant portion of the recording, ApneaLink software (ApneaLink AirTM, ResMed Germany Inc., Martinsried, Germany) scored hypopneas in case of a reduction in airflow of 50% lasting longer than 10 s. Average and lowest blood oxygen saturation (SpO2) were evaluated. (https://airview.resmed.com/resources/welcome-page/pdf/Apnealink-Air_clinical_guide_glo_eng.pdf) Screening results offered by the ApneaLink software were carefully checked by a certified sleep medicine physician with years of experience in sleep apnea screening in the geriatric setting (H.F.) and corrected if necessary using raw data.

#### 2.2.2. Daytime Sleepiness

Daytime sleepiness was assessed by the self-report Epworth Sleepiness Scale (ESS) [16], and the German “Essener Fragebogen Alter und Schläfrigkeit” (EFAS) [17], which is designed as observational scale and completed by the nursing staff. The ESS describes eight activities people frequently engage in, and respondents rate their usual chances of dozing off or falling asleep in these situations on a scale from 0–3. ESS sum scores >10 indicate excessive daytime sleepiness. Since the ESS is not well suited for elderly frail people and people with serious cognitive impairment, we additionally used the German EFAS. The EFAS describes ten situations in which people usually should not be asleep and the nursing staff assessed how often persons were asleep in these situations as well as the severity of daytime sleepiness meaning the degree of impairment in everyday life in these situations on a scale from 0–3. EFAS score is created by multiplying the score of frequency and severity for the item with the highest scores in frequency and severity. EFAS scores >2 indicate at least moderate daytime sleepiness. 

#### 2.2.3. Demographic and Medical Characteristics

Education was assessed by interview of the patient or their legal representatives and classified as primary school, secondary school, or baccalaureate graduation. Information about hypertension, diabetes, hyperlipoproteinemia, smoking, renal insufficiency, coronary heart disease, heart failure, peripheral artery disease, history of stroke, and medication prescription was prospectively collected from patient records. Blood and urine samples were collected, which were analyzed in the hospital’s central laboratory. Noninvasive tests included the measurement of systolic and diastolic blood pressure, and standardized height and weight measurement to calculate body mass index (BMI [kg/m^2^]), overweight being defined as BMI ≥ 30 kg/m^2^.

#### 2.2.4. Cognitive and Emotional Function

Cognitive function was assessed by Mini-Mental state examination test (MMSE) [18], DemTect [19], clock-drawing test [20], Alzheimer’s disease assessment scale–cognitive subscale (ADAS-cog) [21], trail making test (ZVT-G) and figural memory test (FT) from the German “Nürnberger Altersinventar” [22], Timed Test of Money Counting (TTMC) [23], and the German “Alters-Konzentrations-Test” (A-K-T), which measures the ability to concentrate in the elderly [24]. 

Emotional function was assessed by WHO-5 Well-Being Index [25], a score ≤50 indicates reduced well-being and can be regarded as sign of depression [26]. Depression was further assessed by the short form of the Geriatric Depression Scale (GDS) [27], a score ≥6 in the German version being interpreted as a hint towards depression [28].

#### 2.2.5. Mobility

Mobility was assessed by Barthel index, which is a simple clinical index measuring the extent of independence in activities of daily living [29], by instrumental activities of daily living scale, which measures the extent of independence in more complex activities of daily living [30], timed Up & Go, a test of basic functional mobility [31], test of standing balance including side-by-side, semi-tandem and tandem stands [32], Tinetti mobility test, which measures static and dynamic balance [33], walking speed (meter per second during 10 s of walking), hand grip strength of the left and right hand measured with an hydraulic hand dynamometer (Saehan Corporation, Masan, South Korea), and number of frailty criteria fulfilled [34]. 

### 2.3. Statistical Analysis

Continuous data are presented as mean±SD values for normally distributed data and median (Q1; Q3) for non-normally distributed data, categorical data are presented as counts (%). Comparisons between AHI categories (AHI < 5/h, representing absent SDB, 5/h ≤ AHI < 15/h, representing mild SDB and AHI ≥ 15/h representing moderate/severe SDB) regarding demographic data, risk factors and comorbidities, daytime sleepiness, cognitive and emotional function, and mobility were done with (a) one-way ANOVA followed by Bonferroni post-hoc tests for continuous normally distributed data, and (b) Kruskal-Wallis-Test followed by Mann-Whitney-U-Test post-hoc tests for non-normally distributed data, and (c) Chi-square or Fisher’s exact test for categorical data. In a sensitivity analysis, we also used an alternative AHI categorization focusing more on severe SDB (AHI < 5/h, representing absent SDB, 5/h ≤ AHI < 30/h, representing mild/moderate SDB and AHI≥30/h representing severe SDB). Comparisons between patients tolerating the nasal airflow sensor for at least vs less than 6 h were done with (a) *t*-test for continuous normally distributed data, and (b) Mann-Whitney-U-Test post-hoc tests for non-normally distributed data, and (c) Chi-square or Fisher’s exact test for categorical data. Correlation between AHI and MMSE was calculated with Pearson correlation coefficient. *p*-values ≤0.05 indicate statistical significance. All above-mentioned statistics were performed using Statistical Package for the Social Science 22 (SPSS 22) for Windows (SPSS, Chicago, IL, USA). Based on the significant correlation of −0.37 between the Mattis Dementia Rating Scale and the respiratory disturbance index observed by the research group around Ancoli-Israel et al. within the San Diego nursing home studies [10], our power calculation showed that we needed a sample size of n = 52 to detect a correlation between cognitive function and SDB of this strength with a two-sided 5% significance level and a power of 80%. Power calculation was done with G*Power [35].

## 3. Results

In 82 of the 101 dementia patients (81.2%), AHI could be determined during overnight polygraphy according to the manufacturer’s suggestions (https://airview.resmed.com/resources/ welcome-page/pdf/Apnealink-Air_clinical_guide_glo_eng.pdf). Of those 82 patients, 10 patients (12.2%) had an AHI < 5/h, representing absent SDB, 33 patients (40.2%) had a 5/h ≤ AHI < 15/h, representing mild SDB, and 39 patients (47.6%) had an AHI ≥ 15/h representing moderate/severe SDB. Patients in those three AHI categories did not significant differ from each other in demographical and clinical characteristics (Table 1).

Descriptively, patients with an AHI ≥ 15/h more often suffered from heart failure, had lower levels of vitamin D, and were more often women. Even though nearly half of our cohort had at least moderate SDB, levels of daytime sleepiness were rather low with no significant differences between AHI categories (Table 2). 

**Figure 1:**
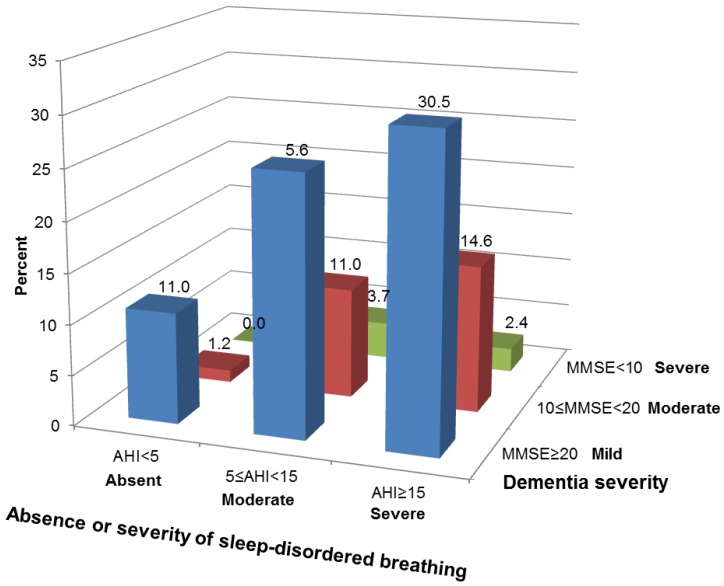
Association between severity of sleep-disordered breathing and severity of dementia. Chi-square test revealed no significant association χ²(4) = 3.33, *p* = 0.521, Cramer‘s V = 0.14. MMSE = Mini-Mental state examination.

With most patients in the study cohort exhibiting mild dementia, there were no significant differences between AHI categories in neuropsychological tests (Table 3). Descriptively, patients with absent SDB showed better performance in the MMSE, DemTect, trail making test, and ADAS-cog memory subscale compared to patients with mild SDB and patients with moderate/severe SDB. When split by SDB severity (AHI < 5/h representing absent SDB, 5/h ≤ AHI < 15/h representing mild SDB, and AHI ≥ 15/h representing moderate/severe SDB) and dementia severity (MMSE ≥ 20 representing mild dementia, 10 ≤ MMSE < 20 representing moderate dementia, and MMSE < 10 representing severe dementia) as it was done in a previous study by Aoki et al. [11],we observed a low to moderate [36] but statistically non-significant association between severity of SDB and severity of dementia (χ²(4) = 3.33, *p* = 0.521, Cramer‘s V = 0.14, Figure 1). Since only few patients in our cohort showed moderate or severe levels of dementia (*n* = 22 with moderate dementia and *n* = 5 with severe dementia), we additionally did a correlation analysis using continuous AHI and MMSE scores, observing a low to moderate association (r = −0.214, *p* = 0.054). 

Since Hoch et al. observed a significant association between SDB severity and dementia severity only in dementia patients without depression but not in patients with both cognitive impairment and depressive symptoms [12], we additionally evaluated the association between SDB severity and dementia severity in non-depressed dementia patients by excluding patients with a geriatric depression score of 6 or more which is in indicator of depressive symptoms [28]. However, also in this subgroup of non-depressed dementia patients we observed no significant association between SDB severity and dementia severity (Table A4).

With overall rather high impairment in activities of daily living and physical functioning including mobility, which represents the typical pattern of geriatric patients, there were no significant differences between AHI categories (Table 4).

Although in our cohort valid AHI measurement was possible in 82 of the 101 dementia patients (81.2%), we observed that acceptance of the nasal airflow sensor of at least 6 h, which is requested for the billing of the diagnostic procedure for health insurance companies in Germany, was present in less than one third of all patients (Table 2). Since literature on the minimal recording time required for valid SDB diagnostics is lacking, we also examined acceptance of the nasal airflow sensor of at least 3 h, which we observed as an acceptable compromise between patient acceptance and still adequate validity for SDB determination and which was already present in half of the study sample. Acceptance of pulse oximetry was higher with about 40% tolerating measurement for 6 h and 60% for 3 h (Table 2). When comparing patients tolerating the nasal airflow sensor for 6 or more hours with those tolerating the nasal airflow sensor for less than 6 h, the tolerant group exhibited a higher frequency of nicotine abuse and coronary heart disease, lower levels of folic acid (Table 5), more symptoms of depression (Table 6), and higher physical fitness (Table 7).

**Figure A1:**
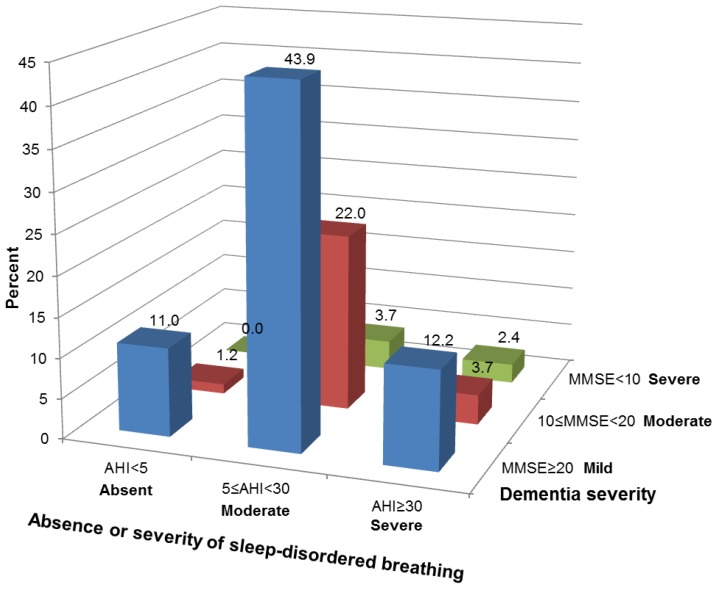
Association between severity of sleep-disordered breathing and severity of dementia. Chi-square test revealed no significant association χ²(4) = 4.67, *p* = 0.313, Cramer‘s V = 0.17. MMSE = Mini-Mental state examination.

In a sensitivity analysis we divided AHI in the alternative categories AHI < 5/h, representing absent SDB, 5/h ≤ AHI < 30/h, representing mild/moderate SDB and AHI ≥ 30/h, representing severe SDB, and observed similar results compared to the previous classification (Table A1, Table A2, Table A3 and Table A5, Figure A1).

## 4. Discussion

In a cohort of 101 consecutive hospitalized geriatric patients mostly exhibiting mild dementia, we observed that the detection of SDB by overnight polygraphy using a portable sleep apnea examination device in the patient room was possible in 81.2% of all patients. About half of all patients with valid SDB evaluation showed at least moderate SDB with about one fifth exhibiting even severe SDB. This prevalence matches the results of a slightly younger American population-based cohort (mean age 73 ± 6 years) which observed a respiratory disturbance index ≥5/h in 81% of the cohort [37] whereas we observed an AHI ≥ 5/h in 88% of our cohort. Further, it matches the prevalence previously observed in elderly dementia patients (80.3 ± 8.6 years) with 89.2% of patients exhibiting a respiratory disturbance index ≥5/h [11]. Despite the high prevalence of SDB in our cohort, levels of daytime sleepiness were rather low and were not significantly associated with SDB severity. In our total cohort, only 7% showed ESS scores >10, which matches previously reported minimal daytime sleepiness levels in people with at least moderate SDB, even though it has to be considered that ESS might not be valid for assessing daytime sleepiness in patients with severe dementia [4].

In line with previous literature [38], we observed that vitamin D deficiency became more pronounced with increasing SDB severity with already moderate SDB patients exhibiting vitamin D deficiency. The mechanisms underlying the relation between SDB and vitamin D level and the direction of effect are however not known so far. The interplay between SDB, cognition and vitamin D is suggested to be further influenced by individual patients’ comorbidity profile. One possible explanation for the positive association between Vitamin D and SDB observed in our study might be that vitamin D levels are reduced by a hypoxia-induced mechanism, since a study in 90 patients with severe obstructive sleep apnea (AHI > 30/h) could show that short-term positive airway pressure therapy was able to recover vitamin D homeostasis in males [39]. Moreover, vitamin D deficiency has been associated with vascular risk factors and events [40], matching our observation of a high stroke and heart failure frequency in patients with moderate or severe SDB which also exhibited vitamin D deficiency. Further studies are needed to determine the clinical relevance of the vitamin D insufficiency observed in SDB patients, especially since the effect of vitamin D on cognition is still not clear with observational cross-sectional and longitudinal data showing that low vitamin D level was associated with worse cognitive performance and cognitive decline whereas intervention studies showed no significant benefit of vitamin D supplementation on cognition [41]. 

Since SDB is often underdiagnosed and instrumental laboratory diagnostics especially of obstructive sleep apnea has been shown to be difficult in dementia patients, we also elucidated which factors influence compliance of nasal airflow sensor as a central part for the detection of obstructive sleep apnea. In our total cohort of geriatric patients with mild dementia, less than one third tolerated nasal airflow sensor diagnostics for 6 or more hours. We observed higher levels of depressive symptoms in those patients tolerating nasal airflow diagnostics for at least 6 h compared to those not tolerating nasal airflow diagnostics for this time period. Higher levels of depressive symptoms could be associated with a higher motivation to tolerate diagnostic procedures in order to detect reasons for reduced emotional well-being and improve emotional well-being. This pattern matches the descriptively higher education level, younger age, higher MMSE scores and higher physical fitness in the tolerant group. Especially when comparing patients where SDB diagnostic was not possible (*n* = 19) with patients where AHI could be determined and which were included in the present analyses of SDB prevalence and its association with cognition (*n* = 82), it has to be noted that patients where SDB diagnostic was not possible had significantly worse cognition (MMSE median = 18.0, Q1 = 14, Q3 = 24) compared with patients where AHI could be determined and which were included in the present analyses (MMSE median = 22.0, Q1 = 19.0, Q3 = 25.0, *p* = 0.034). Thus, the low compliance of SDB diagnostics in our geriatric dementia patient cohort represents a considerable limitation of our study but emphasizes the need for alternative SDB diagnostics in dementia patients. The selective inclusion of patients with higher mental and physical fitness might have biased our results towards a lower prevalence of SDB and a weaker association between SDB and functional patient outcomes such as cognition, emotion and mobility. However, as already shown above, our SDB prevalence was very similar to the prevalence observed by Aoki et al. which was based on a diagnostic procedure tolerated by all patients [11].

In contrast to previous studies, which mostly observed a significant association between SDB severity and dementia severity [10,11,12,13], we only observed a low to moderate statistically not significant association between SDB severity and dementia severity. Reasons for these inconsistent results might be differences in patient cohorts. The research group around Ancoli-Israel et al. for example recruited institutionalized nursing home patients, which had a similar age than our cohort but exhibited severe dementia [10]. Hoch [12] and Reynolds [13] recruited in- and outpatients from a geriatric unit of a psychiatric institute and a geriatric center which again exhibited higher levels of dementia (MMSE = 18) compared to our patients (MMSE = 21). In the most recent analysis, Aoki et al. recruited dementia patients in a psychiatric Japanese hospital, which also exhibited severe dementia (MMSE = 11) but less comorbid conditions since patients with comorbid conditions were not treated in psychiatry [11]. Further, previous studies used less extensive neuropsychological test batteries to assess cognitive function like the Mattis Dementia Rating Scale [10], Blessed Dementia Rating Scale [12,13], or MMSE and Hasegawa dementia scale [11], even though especially vigilance and executive function is influenced by SDB [42]. Our results more resemble the picture seen in healthy elderly cohorts, which also demonstrated no significant association between SDB severity (defined by AHI) and cognitive performance (assessed by a comprehensive neuropsychological test battery) [2].

Due to the cross-sectional design we cannot answer the question whether SDB increases the risk of dementia due to hypoxic brain damage or whether dementia leads to lesions in brain areas associated with breathing, and perhaps both processes take place. Evidence for the mechanism of increases in dementia risk due to hypoxic brain damage exists from (a) preliminary studies showing improvement in cognition in dementia patients with obstructive sleep apnea after positive airway pressure therapy [6], (b) prospective population-based studies showing that SDB predicted incident dementia/cognitive decline in community-dwelling elderly [4,5], and (c) prospective population- based studies showing that SDB increases cardiovascular risk which then mediates increased risk of cognitive decline [43]. Since SDB is often underdiagnosed due to lack of clinical symptoms and lack of tolerance of sophisticated diagnostic procedures including sleep laboratory in the elderly [44], our results in combination with previous literature suggests a potential role for SDB screening in the elderly to uncover a potentially reversible cause of cognitive impairment [45,46]. SDB in the elderly might exert different mechanism than SDB in the middle-aged since elderly, often multimorbid patients, have less reserve to compensate for SDB [9,47]. Especially elderly patients with early dementia pathology might suffer from devastating effects of SDB since additional hypoxemia can aggravate dementia pathology. Detection of SDB in the elderly, especially those suffering from comorbid dementia, is a particular challenge due to reduced acceptance of diagnostic procedures. Although polysomnography in the sleep laboratory with recording of sleep electroencephalography, electrooculography, electromyography, electrocardiography, oronasal airflow, snoring, respiratory effort, oxygen saturation and video of behavior is recommended for SDB diagnosis [15], systems with a reduced number of channels should be used for screening purposes even in ambulatory settings in patients at high SDB risk like in elderly patients with high levels of comorbidity, which are included in our cohort. Positive airway pressure therapy is generally suggested in case of moderate or severe SDB (AHI >15/h) and could be considered in patients with mild SDB (AHI ≤ 15/h) and additionally high cardiovascular risk and/or daytime fatigue also in case of comorbid mild or moderate dementia [48,49]. In our cohort, the majority of patients (84%) showed obstructive sleep apnea and would thus qualify for positive airway pressure therapy. It is estimated that about 15% of Alzheimer’s disease may be attributable to long-term SDB with obstructive sleep apnea being the main risk factor [1,49], suggesting that SDB screening should be incorporated in clinical routine also in the elderly. 

## 5. Conclusions

Sleep-disordered breathing (SDB) is an emerging dementia risk factor but literature on the association between SDB and cognitive impairment is still scarce. For the first time we showed that SDB is highly prevalent in elderly geriatric patients suffering from mild dementia (87.8% with AHI ≥ 5/h), with more than half of these patients exhibiting moderate/severe SDB. We observed a low to moderate association between severity of SDB and severity of dementia. Since preliminary studies already showed that positive airway pressure therapy improved cognition in dementia patients, our data underline the importance of SDB screening in the elderly, especially those with mild dementia. SDB is often underdiagnosed and instrumental laboratory diagnostics and treatment especially of obstructive sleep apnea has been shown to be difficult in dementia patients. We also experienced that less than one third of our cohort of geriatric patients with mild dementia tolerated nasal airflow sensor diagnostic for 6 or more hours. Further studies are needed to develop innovative valid SDB diagnostic and treatment devices which are better tolerated by dementia patients. Additionally, larger studies analyzing the benefit of such innovative SDB screening and subsequent treatment as well as the influence of further comorbidities in geriatric patients have to be conducted. 

## Figures and Tables

**Table 1:** Demographic and clinical characteristics of the study cohort also split by AHI category.

Variables	Total (*n* = 101)	AHI < 5/h (*n* = 10)	5/h ≤ AHI < 15/h (*n* = 33)	AHI ≥ 15/h (*n* = 39)	*p*-Value
Age (years), mean ± SD	84.1 ± 6.5	84.7 ± 8.6	83.5 ± 5.4	84.1 ± 5.8	0.855
Male sex, *n* (%)	32 (31.7)	5 (50.0)	8 (24.2)	14 (35.9)	0.113
Academic degree, *n* (%)					0.289
Primary school	52 (52.0)	6 (60.0)	19 (59.3)	16 (41.0)	
Secondary school	42 (42.0)	4 (40.0)	10 (31.3)	21 (53.9)	
Baccalaureate	6 (6.0)	0 (0.0)	3 (9.4)	2 (5.1)	
Body mass index (kg/m^2^), mean±SD	25.3 ± 5.7	23.9 ± 4.0	25.5 ± 6.1	27.0 ± 5.8	0.251
Overweight, *n* (%)	14 (13.9)	1 (10.0)	3 (9.1)	10 (25.6)	0.205
Smoking, *n* (%)	7 (6.9)	3 (30.0)	1 (3.2)	2 (5.1)	0.101
Systolic blood pressure (mmHg), mean ± SD	140.3 ± 22.1	145.4 ± 20.5	135.1 ± 23.6	140.7 ± 23.7	0.389
Diastolic blood pressure (mmHg), mean ± SD	77.6 ± 34.7	75.9 ± 15.0	72.1 ± 10.9	75.5 ± 13.2	0.471
Antihypertensive medications, *n* (%)	73 (72.3)	6 (60.0)	27 (81.8)	26 (72.2)	0.230
Arterial hypertension, *n* (%)	75 (74.3)	7 (70.0)	28 (84.8)	27 (69.2)	0.281
HbA1c (%), (median [Q1; Q3])	6.8 (5.6; 7.4)	7.1 (6.9; 7.1)	6.9 (5.4; 7.8)	6.5 (5.5; 7.4)	0.649
Antidiabetic medications, *n* (%)	19 (18.8)	2 (20.0)	8 (24.2)	5 (12.8)	0.478
Diabetes mellitus, *n* (%)	28 (27.7	2 (20.0)	11 (33.3)	9 (23.1)	0.684
Hyperlipoproteinemia, *n* (%)	2 (2.0)	0 (0)	0 (0)	2 (5.1)	0.582
Antihyperlipidemic medications, *n* (%)	22 (21.8)	1 (10.0)	8 (24.2)	6 (15.4)	0.265
eGFR (mL/min/1.73 m^2^), mean±SD	58.7 ± 24.6	70.8 ± 22.6	57.3 ± 26.8	59.2 ± 21.9	0.332
Renal insufficiency, *n* (%)	31 (30.7)	1 (10.0)	13 (39.4)	9 (23.1)	0.174
Coronary heart disease, *n* (%)	25 (24.8)	3 (30.0)	10 (30.3)	7 (17.9)	0.424
Heart failure, *n* (%)	31 (30.7)	0 (0)	8 (24.2)	14 (35.9)	0.070
Stroke, *n* (%)	12 (11.9)	0 (0)	2 (6.1)	5 (20.5)	0.540
Peripheral artery disease, *n* (%)	2 (2.0)	0 (0)	1 (3.0)	1 (2.6)	0.999
Anticoagulant medications, *n* (%)	52 (51.5)	8 (80.0)	12 (36.4)	23 (59.0)	0.208
Natrium (mmol/L), mean ± SD	140.0 ± 5.3	143.3 ± 4.3	140.2 ± 5.4	139.7 ± 4.6	0.190
NT-pro BNP (pg/mL), (median [Q1; Q3])	598.3 (266.4; 1357.8)	296.0 (168.8; 631.1)	588.6 (214.0; 1559.5)	633.1 (330.2; 1177.5)	0.280
Vitamin B12 (pg/mL), (median [Q1; Q3])	434.0 (318.0; 645.0)	467.0 (238.5; 860.5)	392.0 (324.0; 710.0)	410.0 (310.5; 614.5)	0.989
Vitamin D (ng/mL), (median [Q1; Q3])	10.8 (6.1; 24.0)	12.0 (7.7; 27.5)	13.5 (8.7; 31.2)	7.9 (5.3; 22.6)	0.082
TSH (mU/L), (median [Q1; Q3])	1.3 (0.9; 2.2)	0.9 (0.8; 1.7)	1.2 (0.9; 2.2)	1.4 (0.8; 1.8)	0.569
Folic acid (µg/L), (median [Q1; Q3])	7.3 (5.2; 10.1)	5.3 (3.9; 11.5)	7.9 (5.4; 10.3)	7.1 (5.3; 9.2)	0.772
Hemoglobin (g/dL), (median [Q1; Q3])	12.2 (10.5; 13.8)	13.0 (10.8; 14.1)	11.0 (10.1; 14.5)	11.6 (10.7; 13.2)	0.426

AHI = Apnea-hypopnea index; eGFR = estimated glomerular filtration rate; HbA1c = Glycated hemoglobin; NT-pro BNP = N-terminal pro-brain natriuretic peptide; TSH = Thyroid-stimulation hormone.

**Table 2:** Sleep-related characteristics of the study cohort also split by AHI category.

Variables	Total (*n* = 101)	AHI < 5/h (*n* = 10)	5/h ≤ AHI < 15/h (*n* = 33)	AHI ≥ 15/h (*n* = 39)	*p*-Value
AHI, (median [Q1; Q3])	14.0 (7.0; 25.3)	3.0 (2.0; 3.0)	8.0 (6.0; 11.0)	26.0 (19.0; 35.0)	<0.001
Nasal airflow sensor ≥6 h, *n* (%)	28 (27.7)	3 (30.0)	10 (30.3)	15 (38.5)	0.761
Nasal airflow sensor ≥3 h, *n* (%)	48 (47.5)	6 (60.0)	20 (60.6)	22 (56.4)	0.950
Pulse oximetry ≥ 6 h, *n* (%)	39 (38.6)	6 (60.0)	17 (51.5)	16 (41.0)	0.477
Pulse oximetry ≥ 3 h, *n* (%)	62 (61.4)	10 (100.0)	23 (69.7)	28 (71.8)	0.131
Average saturation (%), (median [Q1; Q3])	92.0 (90.0; 94.0)	93.5 (89; 95)	92 (90; 95)	92 (90; 94)	0.638
Lowest saturation (%), (median [Q1; Q3])	77.0 (71.0; 82.0)	82.0 (79.8; 82.3)	77.0 (71.8; 82.8)	74 (64.8; 81.3)	0.055
Epworth Sleepiness Scale (score), (median [Q1; Q3])	5.0 (2.0; 8.0)	5.0 (3.0; 8.5)	6.0 (3.0; 8.5)	6.0 (2.0; 8.0)	0.972
“Essener Fragebogen Alter und Schläfrigkeit“ (score), (median [Q1; Q3])	1.0 (0.0; 4.0)	3.0 (0.0; 7.0)	1.0 (0.0; 3.5)	1.0 (0.0; 5.0)	0.515

AHI = Apnea-hypopnea index.

**Table 3:** Cognitive and emotional function also split by AHI category.

Variables	Total (*n* = 101)	AHI < 5/h (*n* = 10)	5/h ≤ AHI < 15/h (*n* = 33)	AHI ≥ 15/h (*n* = 39)	*p*-Value
MMSE (score)	21.0 (17.5; 25.0)	23.5 (20.8; 26.0)	21.0 (16.5; 25.0)	21.0 (19.0; 25.0)	0.203
DemTect (score)	8.0 (6.0; 9.0)	11.0 (7.0; 14.0)	8.5 (7.0; 10.0)	7.0 (6.0; 9.0)	0.060
Clock drawing (score)	4.0 (3.0; 5.0)	3.5 (2.0; 4.3)	4.0 (3.0; 4.5)	4.0 (3.0; 5.0)	0.628
Trial Making Test (s)	90.0 (51.5; 159.8)	73.5 (46.8; 123.5)	82.5 (52.0; 176.3)	85.0 (50.0; 135.5)	0.824
Figural Memory Test (number of correct responses)	7.0 (6.0; 9.0)	8.0 (7.0; 9.5)	6.0 (5.0; 8.3)	8.0 (7.0; 9.0)	0.116
Timed Test of Money Counting (s)	34.0 (13.0; 70.5)	29.0 (6.5; 88.3)	23.0 (12.0; 55.5)	36.0 (15.0; 70.5)	0.510
“Alters-Konzentrations-Test” (number of correct responses)	20.0 (18.5; 20.0)	20.0 (17.8; 20.0)	20.0 (19.0; 20.0)	20.0 (18.5; 20.0)	0.893
WHO-5 Well-Being Index (%-score)	56.0 (40.0; 72.0)	34.0 (19.0; 58.0)	56.0 (38.0; 68.0)	62.0 (44.0; 76.0)	0.065
Geriatric depression scale (score)	3.0 (2.0; 6.0)	5.5 (3.3; 10.5)	3.0 (2.0; 6.0)	3.0 (2.0; 5.0)	0.144
ADAS-cog total (score)	19.0 (13.0; 24.0)	19.0 (12.0; 22.0)	19.0 (12.0; 23.5)	18.5 (13.3; 23.0)	0.859
ADAS-cog memory (score)	12.0 (10.0; 16.0)	14.0 (9.0; 17.0)	12.0 (9.5; 16.0)	11.5 (10.0; 14.0)	0.703
ADAS-cog praxis (score)	5.0 (2.0; 8.3)	5.0 (1.0; 5.0)	5.0 (2.0; 8.0)	6.0 (2.0; 9.0)	0.444
ADAS-cog language (score)	0.0 (0.0; 0.0)	0.0 (0.0; 0.0)	0.0 (0.0; 0.0)	0.0 (0.0; 0.0)	0.860

All data are shown as median with 25th as well as 75th percentile (median [Q1; Q3]). MMSE = Mini-Mental state examination; DemTect = Demenz-Detektions-Test; ADAS-cog = Alzheimer’s Disease Assessment Scale-cognitive subscale.

**Table 4:** Mobility also split by AHI category.

Variables	Total (*n* = 101)	AHI < 5/h (*n* = 10)	5/h ≤ AHI < 15/h (*n* = 33)	AHI ≥ 15/h (*n* = 39)	*p*-Value
Barthel index (score)	47.5 (30.0; 65.0)	60.0 (27.5; 65.0)	50.0 (30.0; 67.5)	50.0 (30.0; 60.0)	0.924
IADL scale (score)	4.0 (2.0; 6.0)	4.0 (1.8; 5.0)	4.0 (3.0; 6.0)	4.0 (3.0; 5.0)	0.335
Timed Up & Go (s)	24.0 (14.0; 39.3)	27.0 (11.5; 92.0)	21.0 (12.0; 31.5)	24.0 (14.0; 39.0)	0.873
Tinetti mobility test (score)	17.0 (11.8; 22.0)	17.0 (13.0; 23.0)	17.5 (12.0; 22.0)	16.0 (8.0; 22.0)	0.821
Test of standing balance (score)	1.0 (0.0; 2.0)	1.0 (0.0; 3.0)	2.0 (0.0; 3.0)	1.0 (0.0; 2.0)	0.814
Walking Speed (m/s)	0.50 (0.30; 0.80)	0.50 (0.35; 1.00)	0.60 (0.33; 0.88)	0.50 (0.30; 0.90)	0.895
Hand grip strength right (kPa)	14.0 (10.0; 21.5)	18.0 (10.0; 31.5)	12.0 (9.0; 20.0)	16.0 (10.0; 22.0)	0.538
Hand grip strength left (kPa)	12.0 (8.0; 20.0)	15.0 (6.0; 27.5)	12.0 (8.0; 20.8)	14.0 (10.0; 20.0)	0.412
Frailty criteria (number)	4.0 (3.0; 4.0)	3.0 (2.3; 4.8)	3.5 (3.0; 4.0)	3.0 (2.0; 4.0)	0.956

All data are shown as median with 25th as well as 75th percentile (median [Q1; Q3]). IADL = Instrumental activities of daily living.

**Table 5:** Demographic and clinical characteristics split by tolerance of the nasal airflow sensor ≥6 h vs. <6 h.

Variables	Nasal Airflow Sensor < 6 h (*n* = 73)	Nasal Airflow Sensor ≥ 6 h (*n* = 28)	*p*-Value
Age (years), mean ± SD	84.6 ± 6.7	82.7 ± 6.3	0.195
Male sex, *n* (%)	22 (30.1)	9 (32.1)	0.845
Academic degree, *n* (%)			0.351
Primary school	41 (56.2)	11 (40.7)	
Secondary school	28 (38.3)	14 (51.8)	
Baccalaureate	4 (5.5)	2 (7.4)	
Body mass index (kg/m^2^), mean ± SD	25.0 ± 5.9	26.2 ± 5.4	0.358
Overweight, *n* (%)	9 (12.3)	5 (17.9)	0.522
Nicotine abuse, *n* (%)	4 (5.5)	3 (10.7)	0.051
Systolic blood pressure (mmHg), mean ± SD	139.5 ± 21.0	142.1 ± 24.9	0.537
Diastolic blood pressure (mmHg), mean ± SD	79.6 ± 44.3	74.6 ± 78.7	0.602
Antihypertensive medications, *n* (%)	53 (72.6)	19 (67.9)	0.958
Arterial hypertension, *n* (%)	55 (75.3)	20 (71.4)	0.872
HbA1c (%), (median [Q1; Q3])	6.9 (5.7; 7.5)	6.8 (5.2; 7.0)	0.333
Antidiabetic medications, *n* (%)	14 (19.2)	6 (21.4)	0.777
Diabetes mellitus, *n* (%)	21 (28.8)	8 (28.6)	0.984
Hyperlipoproteinemia, *n* (%)	2 (2.7)	0 (0.0)	0.999
Antihyperlipidemic medications, *n* (%)	13 (17.8)	5 (17.9)	0.977
eGFR (mL/min/1.73 m^2^), mean±SD	58.4 ± 25.2	59.6 ± 23.5	0.833
Renal insufficiency, *n* (%)	26 (35.6)	6 (21.4)	0.329
Coronary heart disease, *n* (%)	14 (19.2)	11 (39.3)	0.070
Heart failure, *n* (%)	24 (32.9)	7 (25.0)	0.629
Stroke, *n* (%)	10 (13.7)	2 (7.1)	0.505
Peripheral artery disease, *n* (%)	2 (2.7)	0 (0.0)	0.999
Anticoagulant medications, *n* (%)	36 (49.3)	16 (57.1)	0.799
Natrium (mmol/L), mean ± SD	140.1 ± 4.9	140.2 ± 5.5	0.949
NT-pro BNP (pg/mL), (median [Q1; Q3])	627.8 (280.3; 1546.8)	421.7 (236.2; 1173.3)	0.282
Vitamin B12 (pg/mL), (median [Q1; Q3])	433.0 (280.0; 661.0)	408.5 (329.0; 593.5)	0.924
Vitamin D (ng/mL), (median [Q1; Q3])	10.2 (5.6; 25.0)	11.9 (7.7; 17.3)	0.585
TSH (mU/L), (median [Q1; Q3])	1.3 (0.9; 2.2)	1.2 (0.9; 1.8)	0.436
Folic acid (µg/L), (median [Q1; Q3])	8.1 (5.6; 10.9)	6.4 (5.0; 7.7)	0.021
Hemoglobin (g/dL), (median [Q1; Q3])	12.2 (10.5; 13.9)	11.7 (10.6; 13.4)	0.723

AHI = Apnea-hypopnea index; eGFR = estimated glomerular filtration rate; HbA1c = Glycated hemoglobin; NT-pro BNP = N-terminal pro-brain natriuretic peptide; TSH = Thyroid-stimulation hormone.

**Table 6:** Cognitive and emotional function split by tolerance of the nasal airflow sensor ≥6 h vs. <6 h.

Variables	Nasal Airflow Sensor < 6 h (*n* = 73)	Nasal Airflow Sensor ≥ 6 h (*n* = 28)	*p*-Value
MMSE (score)	21.0 (16.5; 24.5)	23.5 (18.3; 25.0)	0.210
DemTect (score)	8.0 (6.0; 9.0)	7.0 (5.0; 9.0)	0.695
Clock drawing (score)	4.0 (3.0; 5.0)	4.0 (3.0; 5.0)	0.983
Trial Making Test (s)	85.0 (55.0; 163.0)	95.0 (45.0; 175.5)	0.670
Figural Memory Test (number of correct responses)	7.0 (6.0; 8.0)	7.5 (5.8; 9.0)	0.928
Timed Test of Money Counting (s)	33.0 (13.0; 61.8)	36.0 (12.0; 90.0)	0.896
“Alters-Konzentrations-Test” (number of correct responses)	20.0 (18.0; 20.0)	20.0 (19.0; 20.0)	0.934
WHO-5 Well-Being Index (%-score)	56.0 (41.0; 76.0)	52.0 (24.0; 64.0)	0.089
Geriatric depression scale (score)	3.0 (2.0; 5.0)	5.0 (3.5; 7.5)	0.004
ADAS-cog total (score)	19.5 (13.0; 25.0)	18.0 (14.5; 22.0)	0.437
ADAS-cog memory (score)	12.0 (10.3; 16.0)	11.5 (9.8; 15.0)	0.404
ADAS-cog praxis (score)	5.0 (2.0; 9.0)	5.5 (2.0; 8.0)	0.613
ADAS-cog language (score)	0.0 (0.0; 0.0)	0.0 (0.0; 0.0)	0.714

All data are shown as median with 25th as well as 75th percentile (median [Q1; Q3]). MMSE = Mini-Mental state examination; DemTect = Demenz-Detektions-Test; ADAS-cog = Alzheimer’s Disease Assessment Scale-cognitive subscale.

**Table 7:** Mobility split by tolerance of the nasal airflow sensor ≥6 h vs. <6 h.

Variables	Nasal Airflow Senor < 6 h (*n* = 73)	Nasal Airflow Senor ≥ 6 h (*n* = 28)	*p*-Value
Barthel index (score)	45.0 (25.0; 61.3)	50.0 (30.0; 68.8)	0.332
IADL scale (score)	4.0 (2.0; 6.0)	3.0 (2.0; 6.0)	0.823
Timed Up & Go (s)	24.5 (14.8; 61.0)	23.5 (12.3; 34.0)	0.358
Tinetti mobility test (score)	16.5 (10.5; 21.0)	17.5 (12.3; 23.5)	0.351
Test of standing balance (score)	1.0 (0.0; 2.0)	2.0 (1.0; 3.0)	0.040
Walking Speed (m/s)	0.60 (0.28; 0.90)	0.50 (0.40; 0.90)	0.813
Hand grip strength right (kPa)	12.5 (9.0; 20.5)	18.0 (12.0; 24.8)	0.014
Hand grip strength left (kPa)	11.5 (8.0; 16.3)	16.5 (10.5; 21.8)	0.022
Frailty criteria (number)	4.0 (3.0; 5.0)	3.0 (3.0; 4.0)	0.391

All data are shown as median with 25th as well as 75th percentile (median [Q1; Q3]). IADL = Instrumental activities of daily living.

## Appendix A

**Table A1:** Demographic and clinical characteristics of the study cohort also split by alternative AHI category.

Variables	Total (*n* = 101)	AHI < 5/h (*n* = 10)	5/h ≤ AHI < 30/h (*n* = 57)	AHI ≥ 30/h (*n* = 15)	*p*-Value
Age (years), mean ± SD	84.1 ± 6.5	84.7 ± 8.6	83.9 ± 5.5	83.3 ± 6.2	0.856
Male sex, *n* (%)	32 (31.7)	5 (50.0)	15 (26.3)	7 (46.7)	0.133
Academic degree, *n* (%)					0.338
Primary school	52 (52.0)	6 (60.0)	28 (50.0)	7 (46.7)	
Secondary school	42 (42.0)	4 (40.0)	23 (41.1)	8 (53.4)	
Baccalaureate	6 (6.0)	0 (0.0)	5 (8.7)	0 (0)	
Body mass index (kg/m^2^), mean ± SD	25.3 ± 5.7	23.9 ± 4.0	25.7 ± 5.7	28.6 ± 6.3	0.105
Overweight, *n* (%)	14 (13.9)	1 (10.0)	8 (14.0)	5 (33.3)	0.221
Smoking, *n* (%)	7 (6.9)	3 (30)	1 (1.8)	2 (13.3)	0.035
Systolic blood pressure (mmHg), mean ± SD	140.3 ± 22.1	145.4 ± 20.5	138.4 ± 22.9	136.9 ± 22.9	0.641
Diastolic blood pressure (mmHg), mean ± SD	77.6 ± 34.7	75.9 ± 15.0	73.4 ± 10.5	75.9 ± 17.7	0.721
Antihypertensive medications, *n* (%)	73 (72.3)	6 (60.0)	40 (70.2)	13 (86.7)	0.317
Arterial hypertension, *n* (%)	75 (74.3)	7 (70.0)	42 (73.7)	13 (86.7)	0.548
HbA1c (%), (median [Q1; Q3])	6.8 (5.6; 7.4)	7.1 (6.9; 7.1)	6.9 (6.0; 7.4)	5.5 (5.5; 7.1)	0.488
Antidiabetic medications, *n* (%)	19 (18.8)	2 (20.0)	13 (22.8)	1 (6.7)	0.421
Diabetes mellitus, *n* (%)	28 (27.7	2 (20.0)	17 (29.8)	4 (26.7)	0.928
Hyperlipoproteinemia, *n* (%)	2 (2.0)	0 (0)	1 (1.8)	1 (6.7)	0.433
Antihyperlipidemic medications, *n* (%)	22 (21.8)	1 (10.0)	10 (17.5)	4 (26.7)	0.390
eGFR (mL/min/1.73 m^2^), mean ± SD	58.7 ± 24.6	70.8 ± 22.6	57.2 ± 24.9	62.2 ± 21.3	0.272
Renal insufficiency, *n* (%)	31 (30.7)	1 (10.0)	19 (33.3)	4 (26.7)	0.404
Coronary heart disease, *n* (%)	25 (24.8)	3 (30.0)	14 (24.6)	3 (20.0)	0.853
Heart failure, *n* (%)	31 (30.7)	0 (0)	17 (29.8)	5 (33.3)	0.102
Stroke, *n* (%)	12 (11.9)	0 (0)	3 (5.3)	4 (26.7)	0.039
Peripheral artery disease, *n* (%)	2 (2.0)	0 (0)	1 (1.8)	1 (6.7)	0.525
Anticoagulant medications, *n* (%)	52 (51.5)	8 (80.0)	27 (47.4)	8 (53.3)	0.350
Natrium (mmol/L), mean ± SD	140.0 ± 5.3	143.3 ± 4.3	140.2 ± 5.1	138.9 ± 4.5	0.129
NT-pro BNP (pg/mL), (median [Q1; Q3])	598.3 (266.4; 1357.8)	296.0 (168.8; 631.1)	617.6 (263.6; 1368.5)	690.7 (295.9; 1289.3)	0.491
Vitamin B12 (pg/mL), (median [Q1; Q3])	434.0 (318.0; 645.0)	467.0 (238.5; 860.5)	392.0 (321.0; 604.0)	400.5 (300.5; 914.5)	0.941
Vitamin D (ng/mL), (median [Q1; Q3])	10.8 (6.1; 24.0)	12.0 (7.7; 27.5)	12.4 (7.2; 26.0)	7.0 (4.6; 11.8)	0.059
TSH (mU/L), (median [Q1; Q3])	1.3 (0.9; 2.2)	0.9 (0.8; 1.7)	1.3 (0.9; 2.2)	1.2 (0.9; 1.8)	0.560
Folic acid (µg/L), (median [Q1; Q3])	7.3 (5.2; 10.1)	5.3 (3.9; 11.5)	7.3 (5.6; 9.5)	7.0 (4.6; 10.2)	0.504
Hemoglobin (g/dL), (median [Q1; Q3])	12.2 (10.5; 13.8)	13.0 (10.8; 14.1)	11.2 (10.2; 13.2)	12.7 (11.1; 14.7)	0.173

AHI = Apnea-hypopnea index; eGFR = estimated glomerular filtration rate; HbA1c = Glycated hemoglobin; NT-pro BNP = N-terminal pro-brain natriuretic peptide; TSH = Thyroid-stimulation hormone.

**Table A2:** Sleep-related characteristics of the study cohort also split by alternative AHI category.

Variables	Total (*n* = 101)	AHI < 5/h (*n* = 10)	5/h ≤ AHI < 30/h (*n* = 57)	AHI ≥ 30/h (*n* = 15)	*p*-Value
AHI, (median [Q1; Q3])	14.0 (7.0; 25.3)	3.0 (2.0; 3.0)	12.0 (8.0; 19.5)	42.0 (33.0; 46.0)	<0.001
Nasal airflow sensor ≥6 h, *n* (%)	28 (27.7)	3 (30.0)	23 (40.4)	2 (13.3)	0.169
Nasal airflow sensor ≥3 h, *n* (%)	48 (47.5)	6 (60.0)	37 (64.9)	5 (33.3)	0.084
Pulse oximetry ≥6 h, *n* (%)	39 (38.6)	6 (60.0)	28 (49.1)	5 (33.3)	0.404
Pulse oximetry ≥3 h, *n* (%)	62 (61.4)	10 (100.0)	39 (68.4)	12 (80.0)	0.078
Average oxygen saturation (%), (median [Q1; Q3])	92.0 (90.0; 94.0)	93.5 (89.0; 95.0)	92.0 (90.0; 94.0)	92.0 (89.0; 93.0)	0.660
Lowest oxygen saturation (%), (median [Q1; Q3])	77.0 (71.0; 82.0)	82.0 (79.8; 82.3)	75.0 (69.0; 82.0)	76.0 (66.0; 81.0)	0.119
Epworth Sleepiness Scale (score), (median [Q1; Q3])	5.0 (2.0; 8.0)	5.0 (3.0; 8.5)	6.0 (2.0; 8.0)	5.0 (2.5; 10.5.0)	0.477
“Essener Fragebogen Alter und Schläfrigkeit“ (score), (median [Q1; Q3])	1.0 (0.0; 4.0)	3.0 (0.0; 7.0)	1.0 (0.0; 4.0)	1.0 (0.0; 6.0)	0.664

AHI = Apnea-hypopnea index.

**Table A3:** Cognitive and emotional function also split by alternative AHI category.

Variables	Total (*n* = 101)	AHI < 5/h (*n* = 10)	5/h ≤ AHI < 30/h (*n* = 57)	AHI ≥ 30/h (*n* = 15)	*p*-Value
MMSE (score)	21.0 (17.5; 25.0)	23.5 (20.8; 26.0)	21.0 (17.5; 24.5)	24.0 (19.0; 25.0)	0.122
DemTect (score)	8.0 (6.0; 9.0)	11.0 (7.0; 14.0)	8.0 (6.0; 9.0)	8.0 (6.3; 9.0)	0.143
Clock drawing (score)	4.0 (3.0; 5.0)	3.5 (2.0; 4.3)	4.0 (3.0; 4.5)	4.0 (3.0; 5.0)	0.669
Trial Making Test (s)	90.0 (51.5; 159.8)	73.5 (46.8; 123.5)	89.0 (50.0; 172.5)	80.0 (52.0; 111.0)	0.808
Figural Memory Test (number of correct responses)	7.0 (6.0; 9.0)	8.0 (7.0; 9.5)	7.0 (6.0; 9.0)	7.5 (7.0; 9.0)	0.266
Timed Test of Money Counting (s)	34.0 (13.0; 70.5)	29.0 (6.5; 88.3)	32.0 (12.5; 70.5)	34.0 (13.5; 42.0)	0.970
“Alters-Konzentrations-Test“ (number of correct responses)	20.0 (18.5; 20.0)	20.0 (17.8; 20.0)	20.0 (19.0; 20.0)	20.0 (20.0; 20.0)	0.318
WHO-5 Well-Being Index (%-score)	56.0 (40.0; 72.0)	34.0 (19.0; 58.0)	56.0 (43.0; 72.0)	62.0 (46.0; 75.0)	0.089
Geriatric depression scale (score)	3.0 (2.0; 6.0)	5.5 (3.3; 10.5)	3.0 (2.0; 6.0)	4.0 (2.0; 5.0)	0.155
ADAS-cog total (score)	20.0 (15.0; 26.5)	20.0 (14.0; 24.0)	20.0 (15.0; 26.3)	21.0 (14.0; 29.0)	0.829
ADAS-cog memory (score)	19.0 (13.0; 24.0)	19.0 (12.0; 22.0)	19.0 (13.0; 23.0)	19.0 (13.8; 25.5)	0.749
ADAS-cog praxis (score)	12.0 (10.0; 16.0)	14.0 (9.0; 17.0)	11.0 (9.0; 16.0)	12.0 (10.8; 14.3)	0.592
ADAS-cog language (score)	5.0 (2.0; 8.3)	5.0 (1.0; 5.0)	6.0 (2.0; 8.0)	5.0 (1.8; 11.3)	0.560

All data are shown as median with 25th as well as 75th percentile (median [Q1; Q3]). MMSE = Mini-Mental state examination; DemTect = Demenz-Detektions-Test; ADAS-cog = Alzheimer’s Disease Assessment Scale-cognitive subscale.

**Table A4:** Cognitive and emotional function also split by AHI category in non-depressed patients (geriatric depression scale score ≤5) *.

Variables	Gesamt (*n* = 69)	AHI < 5 (*n* = 4)	5 ≤ AHI < 15 (*n* = 21)	AHI ≥ 15 (*n* = 29)	*p*-Value
MMSE (score)	21.0 (18.0; 25.0)	22.0 (20.3; 24.5)	22.0 (18.0; 25.0)	21.0 (19.0; 25.0)	0.948
DemTect (score)	7.0 (5.0; 9.0)	8.0 (8.0; 8.0)	8.5 (7.0; 10.0)	7.0 (5.0; 9.0)	0.355
Clock drawing (score)	4.0 (3.0; 5.0)	3 (2.3; 4.5)	3.0 (2.0; 4.0)	3.5 (3.0; 4.8.)	0.490
Trial Making Test (s)	90.0 (53.5; 145.3)	101.0 (52.3; 177.5)	82.5 (50.0; 180.0)	90.0 (51.5; 111.75)	0.867
Figural Memory Test (number of correct responses)	7.0 (6.0; 8.0)	7.0 (5.5; 9.3)	6.5 (5.3; 8.8)	8.0 (6.5; 8.5)	0.438
Timed Test of Money Counting (s)	34.5 (13.8; 63.5)	40.0 (14.0; 40.0)	32.0 (13.0; 51.0)	35.5 (15.0; 102.8)	0.702
“Alters-Konzentrations-Test“ (number of correct responses)	20.0 (18.0; 20.0)	20.0 (17.0; 20.0)	20.0 (18.5; 20.0)	20.0 (19.0; 20.0)	0.996
WHO-5 Well-Being Index (%-score)	64.0 (52.0; 79.0)	54.0 (34.0; 74.0)	56.0 (48.0; 72.0)	68.0 (52.0; 80.0)	0.352
Geriatric depression scale (score)	3.0 (1.5; 4.0)	3.5 (2.3; 4.0)	3.0 (1.0; 3.6)	3.0 (1.0; 4.0)	0.750
ADAS-cog total (score)	22.5 (16.8; 27.5)	24.0 (22.0; 24.0)	20.0 (13.6; 27.0)	23.0 (17.0; 27.0)	0.640
ADAS-cog memory (score)	20.0 (14.0; 24.0)	22.0 (19.0; 22.0)	20.0 (13.0; 23.0)	16.0 (16.0; 24.0)	0.646
ADAS-cog praxis (score)	12.0 (11.0; 16.0)	15.0 (12.0; 16.0)	12.0 (11.0; 16.0)	12.0 (10.0; 15.0)	0.303
ADAS-cog language (score)	6.0 (3.0; 9.0)	5.0 (5.0; 5.0)	5.0 (2.0; 8.0)	7.0 (2.0; 11.0)	0.726

* 8 patients had missing data on the geriatric depression scale. All data are shown as median with 25th as well as 75th percentile (median [Q1; Q3]). MMSE = Mini-Mental state examination; DemTect = Demenz-Detektions-Test; ADAS-cog = Alzheimer’s Disease Assessment Scale-cognitive subscale.

**Table A5:** Mobility also split by alternative AHI category.

Variables	Total (*n* = 101)	AHI < 5/h (*n* = 10)	5/h ≤ AHI < 30/h (*n* = 57)	AHI ≥ 30/h (*n* = 15)	*p*-Value
Barthel index (score)	47.5 (30.0; 65.0)	60.0 (27.5; 65.0)	50.0 (30.0; 67.5)	50.0 (35.0; 60.0)	0.955
IADL scale (score)	4.0 (2.0; 6.0)	4.0 (1.8; 5.0)	3.0 (2.0; 6.0)	4.0 (2.0; 5.0)	0.786
Timed Up & Go (s)	24.0 (14.0; 39.3)	27.0 (11.5; 92.0)	18.5 (12.0; 36.5)	29.0 (24.0; 150.0)	0.089
Tinetti mobility test (score)	17.0 (11.8; 22.0)	17.0 (13.0; 23.0)	17.0 (9.0; 23.0)	15.0 (13.0; 20.0)	0.763
Test of standing balance (score)	1.0 (0.0; 2.0)	1.0 (0.0; 3.0)	2.0 (0.0; 3.0)	1.0 (0.0; 2.0)	0.407
Walking Speed (m/s)	0.50 (0.30; 0.80)	0.50 (0.35; 1.00)	0.55 (0.33; 1.00)	0.50 (0.20; 0.70)	0.481
Hand grip strength right (kPa)	14.0 (10.0; 21.5)	18.0 (10.0; 31.5)	14.0 (10.0; 21.0)	14.0 (10.0; 22.0)	0.767
Hand grip strength right (kPa)	12.0 (8.0; 20.0)	15.0 (6.0; 27.5)	12.0 (8.3; 20.8)	14.0 (10.0; 20.0)	0.854
Frailty criteria (number)	4.0 (3.0; 4.0)	3.0 (2.3; 4.8)	3.5 (3.0; 4.0)	3.0 (2.0; 4.5)	0.976

All data are shown as median with 25th as well as 75th percentile (median [Q1; Q3]). IADL = Instrumental activities of daily living.

## References

[1] Bubu O.M., Brannick M., Mortimer J., Sebastiao Y.V., Wen Y., Schwartz S., Borenstein A.R., Wu Y., Morgan D. (2017). Sleep, Cognitive impairment, and Alzheimer’s disease: A Systematic Review and Meta-Analysis. Sleep.

[2] Sforza E., Roche F., Thomas-Anterion C., Kerleroux J., Beauchet O., Celle S., Maudoux D., Pichot V., Laurent B., Barthelemy J.C. (2010). Cognitive function and sleep related breathing disorders in a healthy elderly population: The SYNAPSE study. Sleep.

[3] Pan W., Kastin A.J. (2014). Can sleep apnea cause Alzheimer’s disease?. Neurosci. Biobehav. Rev..

[4] Yaffe K., Laffan A.M., Harrison S.L., Redline S., Spira A.P., Ensrud K.E., Ancoli-Israel S., Stone K.L. (2011). Sleep-disordered breathing, hypoxia, and risk of mild cognitive impairment and dementia in older women. JAMA.

[5] Blackwell T., Yaffe K., Laffan A., Redline S., Ancoli-Israel S., Ensrud K.E., Song Y., Stone K.L. (2015). Associations between sleep-disordered breathing, nocturnal hypoxemia, and subsequent cognitive decline in older community-dwelling men: The Osteoporotic Fractures in Men Sleep Study. J. Am. Geriatr. Soc..

[6] Ancoli-Israel S., Palmer B.W., Cooke J.R., Corey-Bloom J., Fiorentino L., Natarajan L., Liu L., Ayalon L., He F., Loredo J.S. (2008). Cognitive effects of treating obstructive sleep apnea in Alzheimer’s disease: A randomized controlled study. J. Am. Geriatr. Soc..

[7] Bliwise D.L. (2013). Alzheimer’s disease, sleep apnea, and positive pressure therapy. Curr. Treat. Options Neurol..

[8] Alzheimer’s Disease International World Alzheimer Report 2015: The Global Impact of Dementia. https://www.alz.co.uk/research/world-report-2015.

[9] Young T., Peppard P.E., Gottlieb D.J. (2002). Epidemiology of obstructive sleep apnea: A population health perspective. Am. J. Respir. Crit. Care Med..

[10] Ancoli-Israel S., Klauber M.R., Butters N., Parker L., Kripke D.F. (1991). Dementia in institutionalized elderly: Relation to sleep apnea. J. Am. Geriatr. Soc..

[11] Aoki K., Matsuo M., Takahashi M., Murakami J., Aoki Y., Aoki N., Mizumoto H., Namikawa A., Hara H., Miyagawa M. (2014). Association of sleep-disordered breathing with decreased cognitive function among patients with dementia. J. Sleep Res..

[12] Hoch C.C., Reynolds C.F., Kupfer D.J., Houck P.R., Berman S.R., Stack J.A. (1986). Sleep-disordered breathing in normal and pathologic aging. J. Clin. Psychiatry.

[13] Reynolds C.F., Kupfer D.J., Taska L.S., Hoch C.C., Sewitch D.E., Restifo K., Spiker D.G., Zimmer B., Marin R.S., Nelson J. (1985). Sleep apnea in Alzheimer’s dementia: Correlation with mental deterioration. J. Clin. Psychiatry.

[14] Erkinjuntti T., Partinen M., Sulkava R., Telakivi T., Salmi T., Tilvis R. (1987). Sleep apnea in multiinfarct dementia and Alzheimer’s disease. Sleep.

[15] Berry R.B., Budhiraja R., Gottlieb D.J., Gozal D., Iber C., Kapur V.K., Marcus C.L., Mehra R., Parthasarathy S., Quan S.F. (2012). Rules for scoring respiratory events in sleep: Update of the 2007 AASM Manual for the Scoring of Sleep and Associated Events. J. Clin. Sleep Med..

[16] Johns M., Hocking B. (1997). Daytime sleepiness and sleep habits of Australian workers. Sleep.

[17] Frohnhofen H., Fulda S., Frohnhofen K., Popp R. (2013). Validation of the Essener Questionnaire of Age and Sleepiness in the elderly using pupillometry. Adv. Exp. Med. Biol..

[18] Folstein M.F., Folstein S.E., McHugh P.R. (1975). “Mini-mental state”. A practical method for grading the cognitive state of patients for the clinician. J. Psychiatr. Res..

[19] Kessler J., Calabrese P., Kalbe E., Berger F. (2000). DemTect: A new screening method to support diagnosis of dementia. Psycho.

[20] Shulman K.I., Gold D.P., Cohen C.A., Zucchero C.A. (1993). Clock drawing and dementia in the community: A longitudinal study. Int. J. Geriatr. Psychiatry.

[21] Rosen W.G., Mohs R.C., Davis K.L. (1984). A new rating scale for Alzheimer’s disease. Am. J. Psychiatry.

[22] Oswald W.D., Fleischmann U.M. (1995). Nürnberger-Alters-Inventar.

[23] Nikolaus T., Bach M., Specht-Leible N., Oster P., Schlierf G. (1995). The Timed Test of Money Counting: A short physical performance test for manual dexterity and cognitive capacity. Age Ageing.

[24] Gatterer G. (2008). Alters-Konzentrations-Test.

[25] Staehr Johansen K. (1998). The Use of Well-Being Measures in Primary Health Care–the DepCare Project, World Health Organization, Regional Office for Europe: Well-Being Measures in Primary Health Care–the DepCare Project.

[26] Topp C.W., Ostergaard S.D., Sondergaard S., Bech P. (2015). The WHO-5 Well-Being Index: A systematic review of the literature. Psychother. Psychosom..

[27] Yesavage J.A., Sheikh J.I. (1986). 9/Geriatric Depression Scale (GDS). Clin. Gerontol..

[28] Gauggel S., Birkner B. (1999). Validity and reliability of a German version of the Geriatric Depression Scale (GDS). Z. Klin. Psychol. Psychother..

[29] Mahoney F.I., Barthel D.W. (1965). Functional evaluation: The barthel index. Md. State Med. J..

[30] Lawton M.P., Brody E.M. (1969). Assessment of older people: Self-maintaining and instrumental activities of daily living. Gerontologist.

[31] Podsiadlo D., Richardson S. (1991). The timed “Up & Go”: A test of basic functional mobility for frail elderly persons. J. Am. Geriatr. Soc..

[32] Guralnik J.M., Simonsick E.M., Ferrucci L., Glynn R.J., Berkman L.F., Blazer D.G., Scherr P.A., Wallace R.B. (1994). A short physical performance battery assessing lower extremity function: Association with self-reported disability and prediction of mortality and nursing home admission. J. Gerontol..

[33] Tinetti M.E. (1986). Performance-oriented assessment of mobility problems in elderly patients. J. Am. Geriatr. Soc..

[34] Fried L.P., Tangen C.M., Walston J., Newman A.B., Hirsch C., Gottdiener J., Seeman T., Tracy R., Kop W.J., Burke G. (2001). Cardiovascular Health Study Collaborative Research Group. Frailty in older adults: Evidence for a phenotype. J. Gerontol. A Biol. Sci. Med. Sci..

[35] Faul F., Erdfelder E., Lang A.G., Buchner A. (2007). G*Power 3: A flexible statistical power analysis program for the social, behavioral, and biomedical sciences. Behav. Res. Methods.

[36] Cohen J. (1988). Statistical Power Analysis for the Behavioral Sciences.

[37] Ancoli-Israel S., Kripke D.F., Klauber M.R., Mason W.J., Fell R., Kaplan O. (1991). Sleep-disordered breathing in community-dwelling elderly. Sleep.

[38] Neighbors C.L.P., Noller M.W., Song S.A., Zaghi S., Neighbors J., Feldman D., Kushida C.A., Camacho M. (2018). Vitamin D and obstructive sleep apnea: A systematic review and meta-analysis. Sleep Med..

[39] Liguori C., Romigi A., Izzi F., Mercuri N.B., Cordella A., Tarquini E., Giambrone M.P., Marciani M.G., Placidi F. (2015). Continuous Positive Airway Pressure Treatment Increases Serum Vitamin D Levels in Male Patients with Obstructive Sleep Apnea. J. Clin. Sleep Med..

[40] Beveridge L.A., Witham M.D. (2013). Vitamin D and the cardiovascular system. Osteoporos. Int..

[41] Goodwill A.M., Szoeke C. (2017). A Systematic Review and Meta-Analysis of The Effect of Low Vitamin D on Cognition. J. Am. Geriatr. Soc..

[42] Beebe D.W., Groesz L., Wells C., Nichols A., McGee K. (2003). The neuropsychological effects of obstructive sleep apnea: a meta-analysis of norm-referenced and case-controlled data. Sleep.

[43] Floras J.S. (2018). Sleep Apnea and Cardiovascular Disease: An Enigmatic Risk Factor. Circ. Res..

[44] Lindberg E., Gislason T. (2000). Epidemiology of sleep-related obstructive breathing. Sleep Med. Rev..

[45] Yamout K., Goldstein F.C., Lah J.J., Levey A.I., Bliwise D.L. (2012). Neurocognitive correlates of nocturnal oxygen desaturation in a memory clinic population. J. Clin. Exp. Neuropsychol..

[46] Emamian F., Khazaie H., Tahmasian M., Leschziner G.D., Morrell M.J., Hsiung G.Y., Rosenzweig I., Sepehry A.A. (2016). The Association Between Obstructive Sleep Apnea and Alzheimer’s Disease: A Meta-Analysis Perspective. Front. Aging Neurosci..

[47] Alchanatis M., Zias N., Deligiorgis N., Liappas I., Chroneou A., Soldatos C., Roussos C. (2008). Comparison of cognitive performance among different age groups in patients with obstructive sleep apnea. Sleep Breath.

[48] Mayer G., Arzt M., Braumann B., Ficker J.H., Fietze I., Frohnhofen H., Galetke W., Maurer J.T., Orth M., Penzel T. (2017). German S3 Guideline Nonrestorative Sleep/Sleep Disorders, chapter “Sleep-Related Breathing Disorders in Adults,” short version: German Sleep Society (Deutsche Gesellschaft fur Schlafforschung und Schlafmedizin, DGSM). Somnologie (Berl.).

[49] Netzer N.C., Ancoli-Israel S., Bliwise D.L., Fulda S., Roffe C., Almeida F., Onen H., Onen F., Raschke F., Martinez Garcia M.A. (2016). Principles of practice parameters for the treatment of sleep disordered breathing in the elderly and frail elderly: The consensus of the International Geriatric Sleep Medicine Task Force. Eur. Respir. J..

